# Biofabrication in Congenital Cardiac Surgery: A Plea from the Operating Theatre, Promise from Science

**DOI:** 10.3390/mi12030332

**Published:** 2021-03-21

**Authors:** Laszlo Kiraly, Sanjairaj Vijayavenkataraman

**Affiliations:** 1 Pediatric Cardiac Surgery, Sheikh Khalifa Medical City, Abu Dhabi, United Arab Emirates; 2 Department of Public Health, Semmelweis University, 51900 Budapest, Hungary; 3 Division of Engineering, New York University Abu Dhabi, Abu Dhabi, United Arab Emirates; 4 Department of Mechanical and Aerospace Engineering, Tandon School of Engineering, New York University, Brooklyn, NY 11201, USA

**Keywords:** bioactive molecules, biofabrication, biomaterials, 3D bioprinting, congenital heart disease, congenital cardiac surgery, reconstructive surgery, tissue engineering, translational research, stem cells

## Abstract

Despite significant advances in numerous fields of biofabrication, clinical application of biomaterials combined with bioactive molecules and/or cells largely remains a promise in an individualized patient settings. Three-dimensional (3D) printing and bioprinting evolved as promising techniques used for tissue-engineering, so that several kinds of tissue can now be printed in layers or as defined structures for replacement and/or reconstruction in regenerative medicine and surgery. Besides technological, practical, ethical and legal challenges to solve, there is also a gap between the research labs and the patients’ bedside. Congenital and pediatric cardiac surgery mostly deal with reconstructive patient-scenarios when defects are closed, various segments of the heart are connected, valves are implanted. Currently available biomaterials lack the potential of growth and conduits, valves derange over time surrendering patients to reoperations. Availability of viable, growing biomaterials could cancel reoperations that could entail significant public health benefit and improved quality-of-life. Congenital cardiac surgery is uniquely suited for closing the gap in translational research, rapid application of new techniques, and collaboration between interdisciplinary teams. This article provides a succinct review of the state-of-the art clinical practice and biofabrication strategies used in congenital and pediatric cardiac surgery, and highlights the need and avenues for translational research and collaboration.

## 1. Introduction

Despite significant advances achieved in numerous fields of biofabrication, clinical application of biomaterials (synthetic or natural polymers) combined with bioactive molecules and/or cells (stem cells, autologous adult cells, etc.) in an individualized patient setting largely remains a promise. Three-dimensional (3D) printing and bioprinting evolved as promising techniques used for tissue-engineering, so that several kinds of tissue, e.g., bone, cartilage, skin, liver, heart can now be printed in layers or as defined structures for replacement and/or reconstruction in regenerative medicine and surgery. However, there are still many challenges and requirements, e.g., technological, practical, ethical and legal, etc. aspects that need to be addressed. There is also a wide gap between the research labs and the patients’ bedside. Congenital and pediatric cardiac surgery—mostly dealing with reconstructive and regeneration patient-scenarios—offers itself as a unique avenue for closing the gap in translational research, rapid application of new techniques, collaboration between interdisciplinary teams. In this summary, we present a *status quaestionis*, adding clinical and methodological reflections in which questions are framed today for the clinician and scientist.

## 2. Objectives

- To summarize clinical scene and needs for biomaterials in congenital and pediatric cardiac surgery.
- To present the state of biofabrication relevant to congenital and pediatric cardiac surgery.
- To highlight avenues of translational research and collaboration.

## 3. Scope of the Problem

Congenital heart disease (CHD) comprises a colorful spectrum of anomalies resulting from abnormal development of the heart segments and the great arteries during fetal life. CHD is the most common birth defect and its incidence ranges from 6 to 13 per 1000 live births affecting approximately 1 neonate in every 145 births [1]. It is estimated that about 1,310,000 babies are born with CHD worldwide each year [2]. CHD prevalence has grossly increased as survival to adulthood now reaches 90–95% in developed countries from less than 20% in the presurgical era [3]. In the 2020s, the number of grown-ups living with CHD is expected to surpass the neonatal/infant CHD incidence [4,5]. About 15% of CHD patients who previously underwent cardiac surgery will require further operations (e.g., conduit exchange, valve repairs/replacements, operation for acquired heart disease, etc.). Treating patients with CHD is a commitment for life [6].

Cardiac segments consist of the venous inlet, atria, atrioventricular connections, ventricles, ventriculoarterial connections and the great arteries. Congenital cardiac anomalies present along a wide range of problems in the connections of the cardiac segments, their morphology, their relations, and additional anomalies in any segment [7]. About 66% of congenital cardiac patients need to undergo cardiac surgery; and two-thirds of the surgical patient population require intervention within the first six months of life. Primary complete repair has become a central aim of surgery since the 1980s, where all intracardiac and extracardiac issues would be dealt with by a single-stage operation [8]. Surgical repair may restore the correct plumbing in the minimum range of 0.1 mm, however it has so far been unable to address underlying pathologic/developmental processes that occur in the range of nanometers to micrometers. Intracardiac defects (e.g., atrial septal defect (ASD), ventricular septal defect (VSD)) are closed, connection and/or misalignment problems between segments are corrected (e.g., arterial switch operation for transposition of the great arteries (TGA); repair of tetralogy of Fallot), along with the repair of extracardiac anomalies affecting the great arteries (e.g., hypoplastic aortic arch, missing connection between the right ventricle and pulmonary arteries, etc.) at the same time. In about 30% of all congenital cardiac surgical cases, anomalies cannot be solved by a single operation due to physiological and anatomical reasons [9,10]. These patients should undergo staged-repairs (e.g., univentricular hearts, hypoplastic left heart syndrome, etc.). Congenital cardiac surgery is reconstructive surgery with the aim of restoring biventricular circulation when possible. In the absence of two functionally adequate pumping chamber (ventricle), for the presently lacking clinically applicable regeneration methods [11], univentricular rerouting (Fontan-circulation) remains the only surgical solution [12,13,14].

Contemporary mortality rate of congenital cardiac surgery for neonates, infants, and children is reported between a lower limit of 0.1% (repair of subvalvar aortic stenosis) and an upper limit of 13.2% (Norwood-procedure) [15] that places this human endeavor in the risk-range of Himalayan mountaineering or Space Shuttle flight [16]. Neonates undergoing complex open-heart procedures in high acuity are at high short-term risk. Long-term risk derives from growth and is related to ongoing pathophysiological processes. In the pediatric population, somatic growth and fate of any implanted prosthetic material exerts a significant effect on the consequence and frequency matrix of risk [17]. In the context of biomaterials, consequence is evaluated by quality-of-life metrics, reoperation-free survival, etc., whereas frequency could be referred to as the complication rate of different types of prosthetic material and/or devices.

One of the procedural complexities scoring systems (Aristotle Basic Complexity Score) commonly applied in congenital cardiac surgery enlists 157 primary procedures [18]. Utilization of prosthetic material (e.g., patch, conduit or valve) is involved in half of them (84/156 = 53%), and the likelihood of need for patching and/or conduits increases along with procedural complexity (simplest: 8/34 = 23.5%; simple: 34/69 = 49.3%; complex: 16/25 = 64%; most complex: 27/29 = 93%); X2 (1, N = 157) = 30.68, *p* < 0.001 [19]. Current surgical options using prosthetic material (excepting for defect closure with autologous pericardial patch) have very high failure and reintervention rates particularly in younger patients. Advances in translational research and biofabrication may offer a new option to save these patients from multiple open replacements of traditional biologic tissue valves and conduits [20].

## 4. Biomaterials Clinically Utilized in Congenital Cardiac Surgery at Present

**Figure 1:**
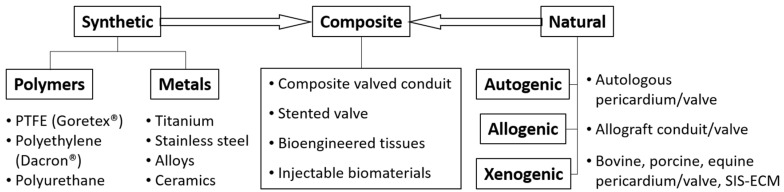
Biomaterials used in congenital and pediatric cardiac surgery. Examples shown herein for illustration and are not all-inclusive. Composite category defines biomaterials that conjoin natural biomaterials with synthetically manufactured scaffolds. Abbreviations: PTFE: polytetrafluoroethylene, SIS-ECM: small intestine mucosa extracellular matrix.

Prosthetic tissues employed in congenital cardiac surgery can be classified as synthetic, natural and/or naturally derived biomaterials and composite (Figure 1).

There are multiple general requirements for biomaterials to fulfil: they need to be biocompatible, and possess physical properties (distensibility, elasticity) to preserve their structural integrity over more than a billion of heart cycles (durability). Physiological properties consist of non-immunogenicity, low thrombogenicity (i.e., ideally no need for anticoagulation and/or additional medication), non-calcification, etc. The dynamic environment of the living organism poises long-term structural resilience on absorbability. Inert biomaterials (e.g., titanium, and to some degree expanded polytetrafluoroethylene, ePTFE) invoke no response but stay as implanted, whereas interactive biomaterials can promote regeneration, remodeling with minimal adverse reactions. Appropriation of the scaffold and seeding it with the recipient’s cells is ultimately dictated by the body’s own (patho)physiological processes. Growth of structures from neonatal to adult sizes adds a new dimension. At present, a neonate with conduit or valve implantation undergoes 2–3 reoperations before reaching adulthood for structural failure or for simply growing out the implant [21].

Practical requirements for biomaterials include easy preparation and/or manufacturing process and handling at a financial affordability. Quick manufacturing of patient-specific prototypes refers to days rather than months as a significant proportion of CHD patients, especially neonates, would require urgent interventions. Non patient-specific (off-the-shelf) implants should be readily available in all sizes. Immune-compatibility and sterility, i.e., no-transmission of infectious agents, substances, is of course of paramount importance. In conclusion, the ideal case scenario, e.g., a living and growing valve prosthesis remains the Holy Grail of congenital cardiac surgery.

Biomaterials in congenital and pediatric cardiac surgery are currently used as (1) patches to close defects, intracardiac (e.g., atrial and/or ventricular, etc.); to create intra/extracardiac tunnels (baffle-procedures); to augment underdeveloped structures; (2) valves, conduits, valved conduits to bridge the gap and/or reconstruct missing segments; and (3) hypothetically to redevelop hypoplastic, or missing chambers and segments. Given the complex and reconstructive nature of the profession, a significant percentage of the procedures apply various biomaterials and positions; e.g., during the complete repair of common arterial trunk, tetralogy of Fallot or transposition of the great arteries with pulmonary atresia, the left ventricular outlet is tunneled towards the aorta with an intracardiac patch, whereas the gap between the right ventricle and pulmonary arteries is bridged with a conduit preferably containing a valve [22].

### 4.1. Patches: Predominantly Non-Cylindrical Reconstruction

Synthetic polymers like expanded polytetrafluoroethylene (ePTFE, Goretex^®^, W. L. Gore and Associates, Newark, DE, USA), and polyethylene terephthalate (PET, Dacron^®^, DuPont, Kinston, NC, USA) have long been used with significant success [21]. They are biocompatible, and their biomechanical properties are adequate for employing them as patches in closing defects, creating baffles, augmenting outflow tracts and extracardiac vessels, etc. Dacron stimulates inflammatory reaction and fibrosis, whereas ePTFE is almost ‘invisible’ to the host organism. Its minimal foreign-tissue reaction also makes it vulnerable to infective endocarditis and to a slightly increased incidence of patch dehiscence [23]. The latter aspect could also be attributed to a structurally limited ability for stretching and remodeling that also translates into persistent stitch-hole bleeding in high-pressure arterial reconstructions. Synthetic patches—especially ePTFE—rarely calcify; their major drawback is their inability to grow, and loss of pliability over time [21].

Based on their strength and biocompatibility, chemically non-reactive metals (titanium, stainless steel, gold) have been used as stents, defect closure devices for several decades. They can be deployed via catheter-based procedures to open the lumen and/or keep it open as in the case of vascular stents (e.g., pulmonary artery, venous, right ventricle outflow tract (RVOT), patent arterial duct (PDA)) or devices to close intra- and extracardiac defects (e.g., ASD, VSD, major aortopulmonary collaterals arteries (MAPCAs), PDA, etc.). Some stents are re-dilatable to follow growth [24], and they may contain a composite valve (e.g., Melody^TM^ transcatheter pulmonary valve, Medtronic, Minneapolis, MN, USA).

**Figure 2:**
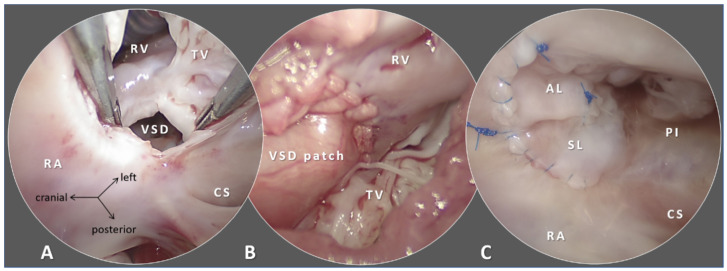
Intraoperative endoscopic images of ventricular septal defect (VSD) patch closure during repair of tetralogy of Fallot. (**A**) The septal leaflet (SL) of the tricuspid valve (TV) is detached and retracted and the VSD is assessed. The defect has fibrous ridges. (**B**) Left ventricle to aorta tunnel is completed by autologous pericardium patch, thus the VSD is closed (transventricular view). (**C**) The detached tricuspid leaflets are reattached to annulus and commissuroplasty is performed. Abbreviations: AL: anterior leaflet of the tricuspid valve; CS: coronary sinus; PI: posteroinferior leaflet of the tricuspid valve; RA: right atrium; RV: right ventricle; SL: septal leaflet of the tricuspid valve; TV: tricuspid valve.

Autologous pericardium has been the first-choice patch material in congenital cardiac surgery [25]. Autologous pericardium is readily available (at primary operations), inexpensive, offers exceptional handling characteristics, is non-thrombogenic and naturally resists infection [26]. Free patches of the parietal pericardium are typically utilized. Histologically, pericardium is a transport membrane formed by mesothelial cells (innermost layer), collagen bundles with interspersed scant elastic fibers (fibrosa) and it is not designed to withstand high transmural pressures. In reacting to extra-physiological stimuli, fibroblast proliferation followed by deposition of granulation fibrous tissue in the extracellular matrix occurs [27]. Both shrinkage and aneurysm formation of the pericardium patches has been observed in extracardiac [28,29,30] and intracardiac [31,32,33] repairs. Cross-linking the collagen fibers with glutaraldehyde increases the strength of the biomaterial; however, glutaraldehyde processing introduces calcification [34,35] (Figure 2).

Availability of autologous pericardium may be insufficient at reoperations, thus allotropic (allograft patches, see below) and xenotropic (bovine, porcine pericardium) decellularized, non-immunogenic biomaterials have widely been employed. Bovine, porcine pericardium has higher layered structural protein content that results in a more robust (stiffer and thicker) prosthetic material still with preserved elastic properties [36]. However, there is higher degree of inflammatory response, cytotoxicity, and calcification [37] experienced with xenograft patches, not fully resolved with glutaraldehyde treatment [38,39]. Thus, numerous technologies have been introduced for removing cells and preserving the original extracellular matrix (decellularization) [40], and for anti-calcification and lessening cytotoxicity [41,42]. Currently bovine, porcine pericardium is the choice of patching material whenever autologous pericardium is unavailable or unsuitable. We refer to the use of porcine small intestine submucosa extracellular matrix (SIS-ECM) under biodegradable materials (see Section 4.4).

Cryopreserved allograft patches are more expensive and could add inconvenience in handling (i.e., special transport, storage and thawing arrangements are required) compared to off-the-shelf xenografts. However, this patch material has remained the preferred choice in Norwood-arch repair for its concave geometry and hemostatic properties [43]. Below 1 year of age, allograft calcification is accelerated [44] that may point to immunological processes [45]. Furthermore, higher panel-reactive antibody levels persist for years after allograft implantation [46] that could jeopardize later pediatric cardiac transplantation in these patients [47,48].

The growth aspect of the intracardiac patches does not seem to be a major issue as neo-intima formation covers the prosthetic material; the heart remodels and grows around the mostly encapsulated patch. Current techniques of intracardiac tunneling aim to create straight flow pathways, thus, reoperations from lack of growth of an intracardiac patch are extremely rare [49]. Similarly, extracardiac vessels augmented with a prosthetic patch can grow provided sufficient native tissue is available. Despite occasionally complex geometry of intracardiac/extracardiac patches, the patch itself exhibits 2D characteristics and the availability of native tissue components provides the third dimension required for growth.

### 4.2. Conduits, Valved-Conduits: Cylindrical Reconstructions

**Figure 3:**
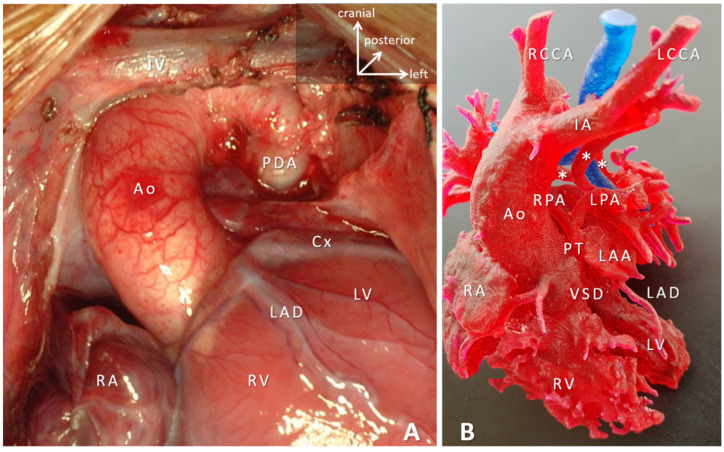
(**A**) Intraoperative representation of pulmonary atresia with absent intrapericardial pulmonary arteries. Ascending aorta (Ao) is the only outlet from the heart. Patent arterial duct (PDA) perfuses the left pulmonary artery (not visible). Left atrial appendage is retracted to expose the left coronary artery system (LAD: left anterior descending and Cx: circumflex branches). (**B**) 3D-printed blood-volume model of pulmonary atresia, VSD and major aortopulmonary collaterals arteries (MAPCAs); right aortic arch. The right ventricle outflow tract is missing and the native pulmonary arteries are hypoplastic. Majority of the pulmonary circulation depends on the MAPCAs (*). Surgical planning is grossly supported by a detailed 3D-printed models. In both cases, the paramount surgical task involves reconstruction of the intrapericardial pulmonary arteries and connecting them to the right ventricle via a preferably valved and growing conduit. Abbreviations: AO: ascending aorta; Cx: circumflex branch of the left coronary artery; IA: innominate artery; IV: innominate vein; LAA: left atrial appendage; LAD: left anterior descending branch of the left coronary artery; LCCA: left common carotid artery; LPA: left pulmonary artery; LV: left ventricle; PDA: patent arterial duct; RA: right atrium; RCCA: right common carotid artery; RPA: right pulmonary artery; RV: right ventricle; VSD: ventricular septal defect. Trachea is shown in blue.

Conduits are utilized to bridge the gap or missing segment (e.g., pulmonary atresia), to bypass a lesion (e.g., aberrant coronary artery in the right ventricle outflow tract); to replace pathologic tissue (e.g., in Marfan-syndrome), to create arteries (e.g., common arterial trunk). Valves are included to protect ventricular function (e.g., reoperation after tetralogy of Fallot repair with transannular patching resulting in pulmonary regurgitation) (Figure 3).

**Figure 4:**
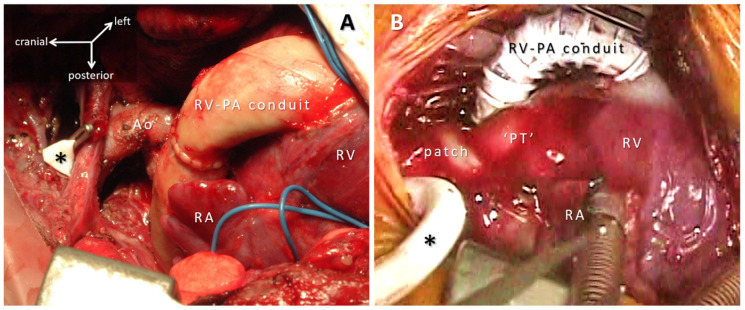
Various biomaterials utilized during complex neonatal cardiac reconstructive surgeries. Images taken from the intraoperative video recording. (**A**) Right ventricle-to-pulmonary artery (RV-PA) connection with valved bovine conduit. (**B**) RV-PA connection with ringed expanded polytetrafluoroethylene (ePTFE) conduit; the pulmonary trunk serves as the systemic outlet of the heart and it is connected to the aortic arch, ascending and descending aorta in a complex anastomosis augmented with autologous pericardium patch. During the arch repair, selective cerebral perfusion is performed through the ePTFE tube (*) connected to the innominate artery. Abbreviations: Ao: ascending aorta; PT: pulmonary trunk; RA: right atrium; RV: right ventricle.

Closing the circle by implanting a cylinder instead of a patch adds extra problems of anisotropy and the growth aspects of circumferential prostheses. Synthetic tubes (ePTFE) as a system pulmonary shunt [50] and/or Sano RV-PA conduit [51] are implanted to allow regulated flow on a temporary basis, so growth potential is not considered (Figure 4). Adult size ePTFE tubes are implanted on the venous side in extracardiac total cavopulmonary connection where the issue of anisotropy is circumvented by the lack systolo-diastolic pulsatility [52]; smooth geometry of the ePTFE extracardiac conduit preserves kinetic energy and contributes to low thrombogenicity [53,54].

**Figure 5:**
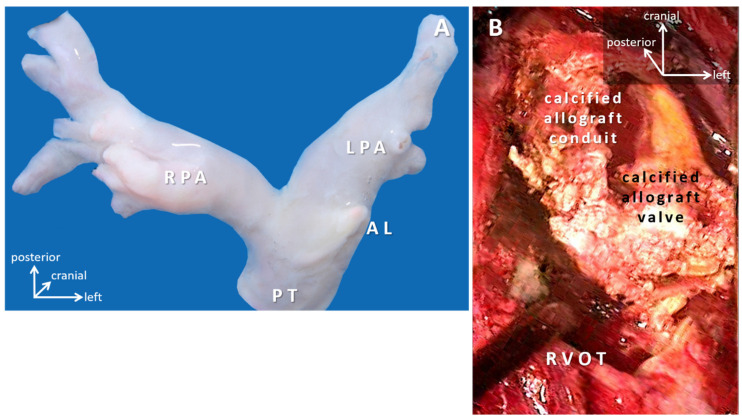
(**A**) Pulmonary allograft at implantation. (**B**) Intraoperative image of severely calcified allograft conduit. Abbreviations: AL: insertion point of the arterial ligament; LPA: left pulmonary artery; PT: pulmonary trunk; RPA: right pulmonary artery; RVOT: right ventricle outflow tract.

Human valved aortic and pulmonary trunk allograft conduits were introduced in the 1960s [55], and have remained the gold-standard for comparing performance [56]. Early devastating graft failure associated with formaldehyde fixation prompted less-aggressive preservation methods and cryopreservation that focus on fibroblast (featuring no human leukocyte antigen (HLA) Class I antigens) viability. Fibroblasts nurture the scaffold of elastic/collagen membranes that when unfolded presents a nidus for calcification. Calcification is aggravated by inflammatory responses, halted growth potential, and results in shrinkage of the conduit [57]. Valvar leaflets remain mobile and free of calcification for a long period [56]. Late failure is signified by leaflet calcification, fibrosis, and degeneration, but no inflammation [58], whereas, rapid failure plus lymphocytic infiltration in valve leaflets and aortic sleeves is consistent with rejection [59]. Degradation of allografts in younger children seems accelerated [60]. Availability of allografts is limited in smaller diameters; they also require special storage, transport and thawing arrangements [61] (Figure 5).

Xenotropic (bovine, porcine) valved conduits offer an off-the-shelf advantage in all sizes, excellent handling properties, and competitive follow-up results for RVOT reconstruction. There are conflicting reports regarding their performance and durability; most studies found good early and mid-term conduit function but also increasing occurrence of premature valve incompetence, aneurysm formation, and supravalvular fibrotic stenosis [62]. A Contegra^®^ bovine jugular vein valved conduit (Medtronic, Minneapolis, USA) is commonly implanted with an acceptable medium-term record [63]. Stenosis of the distal anastomosis frequently occurs that distends the conduit and renders its valve regurgitant [62,64]. Other models with very high early failure rate had been withdrawn from the market [65]. A smaller conduit diameter (~12 mm) and younger age (less than 1 year of age) are predictors for early graft dysfunction and reinterventions [64]. Apparently, none of the currently available allograft or xenograft conduits grows, so the recipients of these implants are subscribed to further interventions [66].

### 4.3. Valve Replacement and Valve Reconstruction in Patients with Somatic Growth

Cardiac valve replacement has a history of over 60 years. Mechanical valves excel with their endurance. In valve leaflets made of pyrolytic carbon, the sewing ring is covered with polyethylene terephthalate or PFTE that have low thrombogenicity; however, a general consensus exists that mechanical valves still require lifelong anticoagulation with vitamin-K antagonists [67,68]. Need of adherence to strict anticoagulation protocols is a major quality-of-life drawback for a growing child, especially in underprivileged circumstances. Furthermore, the current design of the hinging mechanism translates into high inertia and integral transvalvular pressure gradient towards the smaller diameters (<17 mm). Biological valves having bovine or porcine pericardium leaflets mounted on a supporting pyrolytic carbon and titanium frame/stent do not need long-term anticoagulation, however, they have a reduced lifespan [69]. There is no uniform consensus in labelling of valve prostheses’ size, in vivo and in vitro testing and reporting of the haemodynamic performance and thrombogenicity that makes the comparison of the various valve models difficult [70]. The opening and closing mechanism of both mechanical and stented biological valves is different [71] from the physiological process based on currents returning along the wall of aortic sinuses, described by Leonardo da Vinci in 1513 [72]. A pulmonary autograft (Ross-procedure) contains native leaflets along with their sinuses and it offers superb haemodynamics, proven growth potential and no thrombogenicity. Despite having an autologous and viable valve replacement, post-operative Ross-patients still face late reoperations for autograft dilation [73], although at a lesser rate than that for the replacement of the RV-PA conduit [74]. Apparently, no other currently available prosthetic valve has growth and/or regeneration potential [75].

In the absence of a growing cardiac valve prosthesis, an emphasis has been put on valve repair and reconstructions in congenital cardiac surgery [21,76]. We only address here aspects related to biomaterials utilized in various clinical situations. Excepting for decellularized allografts, porcine small intestinal submucosa and the synthetic polymers, all other currently available products are treated with glutaraldehyde which can promote calcification and halt cellular ingrowth from the recipient [77]. Xenotropic tissues can provoke inflammatory response leading to early degeneration as mentioned earlier. Various techniques and prosthetic materials have been used for semilunar valve repair with encouraging mid-term results in the adolescent age group [78,79,80]. Unfortunately, valvuloplasties are renowned for failing within 5–15 years [81].

Reconstruction of the RVOT with monocusp valve leaflet inclusion and transannular patch in tetralogy of Fallot repair can be regarded as a special case of valve repair [82]. The monocusp valve—in accordance to Leonardo’s theory—is closed by the blood current returning from the pulmonary trunk, where the proximal segment of the transannular patch is analogous to the corresponding sinus of Valsalva. The concept offers advantages in the early postoperative period to preserve right ventricular function and thus may allow earlier repair [83]. With glutaraldehyde fixation, strategic positioning and limiting the size of the autologous pericardium transannular patch there has been minimal incidence of aneurysm formation [84]. The monocusp leaflet, however, typically becomes immobile independent of the prosthetic material (e.g., 0.1 mm ePTFE or autologous pericardium is used) on long-term [85]. There are currently various tissue-engineering attempts to develop viable implants focusing on rheological properties, structural and viability aspects [86,87,88,89].

### 4.4. Biodegradable Devices: Disappear Once Their Job Is Done

Most of the biomaterials currently available in pediatric cardiac surgery are inert, e.g., intracardiac patches are covered with neointima, thus their surface is neither exposed nor interact; conduits stay in place until they degrade by outgrowth-shrinkage (interrelated), calcification, and/or immunological reaction. Valves succumb to structural failure e.g., cusp tears, pannus growth, pseudoaneurysm formation, disintegration by infective endocarditis, valve thrombosis and other factors specific to valve type [69].

Interactive biomaterials, on the other hand, combine a dynamic role of a structurally strong scaffold and an integrative platform for regeneration processes of missing/hypoplastic segments and/or damaged structures [88]. In view of the long-term implication of pediatric cardiac repairs, interactive biomaterials should intrinsically remodel and/or absorb in interaction with the surrounding tissue, while responding to physiologic changes and somatic growth. Scaffolds provide structural integrity and support recipient cells, supply them with nutrients and control cell behavior [90]. Dynamic balance between diminishing mechanical properties of the scaffold and its appropriation by recipient’s tissues and cells is preferably determined by timely metabolic rather than inflammatory processes [91].

The SIS-ECM is a naturally derived biomaterial obtained after removing the mucosa, serosa, and muscle layers from the small intestine of pigs. SIS-ECM as an absorbable biomaterial was evaluated as scaffold for myocardial regeneration [92] and patching procedures [93]. SIS-based scaffolds failed in some in vivo studies for the inability to amend their degradation rate, the possibility of immune reactions and technological complications [94].

Implantable occluder- and stent-devices made of polylactic-polyglycolic acid, polydioxanone gradually absorb within 2–3 years. These polyesters degrade in aqueous solutions spontaneously by hydrolysis. The scaffolds are biocompatible, degradation rate is alterable, and they are easy to construct, so they hold the promise for becoming candidates for pediatric cardiac applications [95].

Biodegradable implants conform to the (e.g., Confucian) idea of body integrity without a foreign material. The concept of ‘implantless’ defect (ASD, VSD, etc.) closure, bioabsorbable conduits and valves support cultural sensitivity and may be better accepted [96].

## 5. Biofabrication for Congenital Cardiac Surgery

Congenital cardiac surgery extensively involves use of patches, conduits, and valves to establish anatomic continuity and functional restoration as discussed in the previous sections [97]. The limitations associated with xenografts, allografts, autografts, and other mechanical prosthetics include but are not limited to immune responses and rejection, donor site morbidity, limited availability, reduced durability, lack of growth potential, and increased risk of infection and thrombosis, eventually leading to increased rate of morbidity and mortality during or after the surgery. Biofabrication strategies to produce biomimetic acellular or cell-laden constructs had been explored as promising techniques to overcome the aforementioned challenges of traditional surgical materials and devices [91,92,93].

Biofabrication strategies for congenital cardiac surgery could be classified into two categories: acellular constructs and cell-laden constructs. Acellular constructs include anatomical models for surgical planning and acellular valves, patches, and conduits, mainly providing structural support or anatomic continuity for normal functioning of the heart. Cell-laden constructs, on the other hand, contain viable living cells, preferably patient-derived. Both acellular and cellular constructs are primarily made out of polymeric biomaterials (natural, synthetic, or a composite of both), while other materials such as metals (predominantly in stents and mechanical valves) are also used [98].

### 5.1. Acellular versus Cellular Constructs

One of the main advantages of acellular constructs is the ease of clinical translation. Risks associated with the use of cells, patient-derived or other origins, are completely avoided. Other advantages include ease of storage and maintenance, lesser lead time for manufacturing, and reduced immunological risks. The major disadvantage of acellular constructs lies in the lack of growth potential and potential degradation without tissue restoration.

Cellular constructs are ideal in a clinical setting, where an engineered tissue fabricated using biocompatible materials and patient-derived cells would go a long way in functional restoration of normal cardiac functions. While there are risks associated with the use of cells and the myriad challenges for clinical translation [99], it is still being perceived as the best approach for tissue repair and replacement.

Given the pros and cons of acellular and cellular constructs, the ideal approach has to be determined based on the type and condition of CHD, demography, comorbidities, and other cultural factors. For example, in the case of cardiac patches, where the current gold standard is autologous pericardium or cryopreserved allografts, engineered acellular patches that could be manufactured as off-the-shelf products could be ideal while cellular conduits that has the potential to integrate and grow with the patient becomes an ideal approach for pulmonary atresia. The ideal approach should be adopted based on close discussions between the surgeon, the engineer, and the patient (Table 1).

### 5.2. Tissue Engineering and Bioprinting

Tissue engineering is an interdisciplinary field dealing with the fabrication of cellular constructs, tissues, and organs. A traditional tissue engineering approach involves fabrication of a biomimetic scaffold (using various scaffold fabrication techniques such as melt molding, solvent casting, freeze-drying, gas foaming, electrospinning and more recently 3D printing) [100], seeding of cells (autologous cell lines or stem cells), and maturation in a bioreactor [101]. Additive manufacturing or 3D printing, more specifically bioprinting, has revolutionized the field of tissue engineering by enabling the biomimicry of the native tissue architecture. Replication of the complex cell-tissue hierarchy is possible with bioprinting.

**Figure 6:**
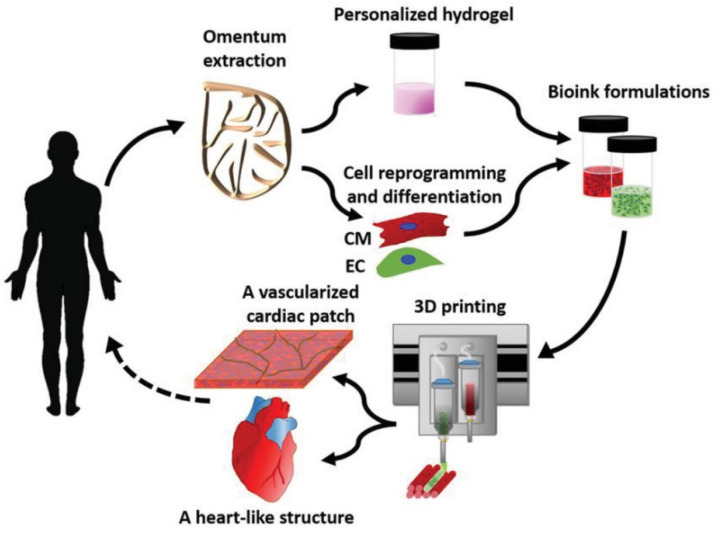
Concept schematic of bioprinting a patient-specific personalized cardiac tissue [112].

Many successful attempts of bioprinting cardiac tissues have been reported in the literature. These include cardiac patches [102,103,104,105], valves [106,107,108], blood vessels [109,110,111], and entire bioartificial hearts [112]. The recent work on bioprinting a bioartificial heart followed an ideal approach (Figure 6) of harvesting omentum tissue from the patient; cells are isolated from the extracted tissue while the matrix is used for formulating a personalized hydrogel. The hydrogel is then suspended with the cells (after reprogramming or differentiation as required) to formulate the bioink, which is processed using a bioprinter to fabricate the cardiac tissue with the intended design. While the process is straightforward, there are several challenges before it could become a standard clinical procedure. Some of those challenges and potential solutions are presented in the sub-sections below.

#### 5.2.1. Replicating Biomimicry

There were many successful reports on replicating the structural biomimicry of cardiac tissues. For instance, Capulli et al. [113] devised an electrospinning process to fabricate fibrous heart valve scaffolds that mimicked the native leaflet fibrosa in terms of the multiscale architecture and mechanical properties. Several aspects of the native leaflet architecture were replicated including the circumferential fiber orientation to withstand the transvalvular loading during diastole and a non-woven mesh structure that allow elastic, radial stretching during systole. There are several other studies that accomplished the structural biomimicry as well such as the off-the-shelf decellularized tissue-engineered heart valve (TEHV) made of biodegradable synthetic materials and vascular-derived cells that fared well with homograft controls [114,115,116]; and the elastomeric scaffolds with curvilinear fiber orientations mimicked key microstructural features of the native leaflet [117]. While these studies could reproduce the structure of the native cardiac tissue, the functional biomimicry is not fully imitated. Despite numerous in vivo studies demonstrating satisfactory restoration of the mechanical function (systolic and diastolic movements with optimal blood flow) involving these scaffolds/valves, long-term in vivo functionality with ample evidence of promoting native tissue regeneration or cellular remodeling is lacking [113]. To this end, proper selection of (stem cells or differentiated) cells and incorporation of biochemical cues such as growth factors becomes necessary. The current approaches in achieving the biomimicry are isolated in terms of structural and functional biomimicry. There has to be a focused effort from a multi-disciplinary team to define and achieve a complete biomimicry both in terms of structure and function.

#### 5.2.2. Cells

While the adult, mature, differentiated myocardial cells are much easier and less risky to use than stem cells, they possess the disadvantage of reduced viability and proliferation [118,119]. In addition, their limited availability and the need for a highly invasive procedure to obtain these cells makes their use practically infeasible and advocates stem cells [120]. Stem cells offer the advantage that they could differentiate into different cell lineages depending on the microenvironment, growth factors, and nature of the substrate. Cardiac tissue contains many different cell types, for example, a fully differentiated adult human ventricular myocardium has 33% cardiomyocytes (CMs), 24% endothelial cells (ECs), and 43% other cells including cardiac fibroblasts (CFs) [121]. Providing a biomimetic cell microenvironment for the stem cells would ensure proper differentiation into specific lineages.

Although there are many different stem cell types and sources such as embryonic stem cells (ESCs) and bone marrow-derived stem cells (BMSCs), the use of umbilical cord and placental stem cells (UCSCs) is a potential choice for congenital cardiac surgery. Umbilical cord, umbilical cord vein, placenta, and Wharton’s jelly are rich sources of multipotent stem cell populations [122]. There are several advantages of UCSCs. Being ethically non-controversial and relatively inexpensive, the procedure for UCSCs collection is also simpler and causes no pain, i.e., autologous tissue is readily available at delivery of the baby [123]. Stem cells can be cryopreserved and stored for later application [124]. The greatest advantage lies in the greater tolerance of HLA mismatches than other stem cell types such as BMSCs, opening the possibilities of using UCSCs allogenically and as off-the-shelf sources [125]. Despite the very many advantages of UCSCs, there are only a few studies reported on the use of UCSCs for cardiac tissue engineering applications. These include the studies by Kim et al. [126] on the effect of transplanting UCSCs in four patients with Buerger’s disease and Ichim et al. [127] on the use of intravenously administered allogeneic placental matrix-derived mesenchymal stem cells for treatment of dilated cardiomyopathy. Transplantation of UCSCs in the former study [126] resulted in disappearance of ischemic rest pain, increased number and size of digital capillaries, reduced vascular resistance and improved peripheral circulation; the latter study [127] reported clinical improvement in dilated cardiomyopathy. UCSCs have been less explored in cardiac tissue engineering and cardiovascular regeneration so far [128] but have a huge potential to be explored in the future.

In addition to UCSCs, induced pluripotent stem cells (iPSCs) also can be explored for use in cardiac tissue engineering and bioprinting [120]. There were several studies published on successful differentiation of iPSCs to cardiomyocytes and more recently to subtype-directed differentiation of human iPSCs to atrial and ventricular cardiomyocytes [129]. Somatic cells such as fibroblasts or keratinocytes that are easy to obtain could be reprogrammed into iPSCs, which could then be differentiated into cardiomyocytes or other cell lineages [120]. However, the inherent challenges associated with the reprogramming of somatic cells into iPSCs and requirement of excellent purification systems [130] limit their widespread use at present.

#### 5.2.3. Materials

Advanced tissue engineering tools such as 3D bioprinting could furnish the structural biomimicry and stem cells could help in restoring the native tissue function but the success of both depends on the right choice of materials. Polymeric materials are predominantly used for engineering cardiac tissues due to their excellent biocompatibility and the ability to tune their composition to obtain a range of physical, chemical, and mechanical properties. Both naturally derived polymers (e.g., collagen, gelatin, chitosan, fibrin, alginate, etc.) and synthetic polymers (e.g., polycaprolactone (PCL), polylactic acid (PLA), polyglycolic acid (PGA), etc.) are used. Each group has its pros and cons as summarized in Table 2. Hybridization of natural and synthetic polymers can beneficially combine their respective advantages while minimizing the weaknesses [131].

Despite several successes in the synthesis and proof-of-concept studies on formulating a suitable ‘bioink’ for bioprinting of cardiac tissues, the ideal cardiac bioink with optimal physio-chemical-mechanical properties to control the cardiac niche for appropriate post-printing stem cell differentiation has not been defined yet [119,132,133]. The reason lies in the associated expectations of such an ideal bioink, which includes non-immunogenicity, paracrine signaling, and optimal stiffness besides being biocompatible and biodegradable. Cell-spheroid bioinks which are scaffold-free, self-sustainable cell aggregates have been used with considerable success [134], but many optimization and long-term in vitro and in vivo studies specific to cardiac tissue are still required. Polymers/hydrogels are just one component of the bioink, the others being cells and growth factors. A proper cardiac cell ratio has to be established as there is no universally accepted ratio [119]. One study [135] reported a ratio of primary adult CMs: ECs: CFs-1:3:6 as optimal and another study [136] reported a ratio of 2:1:1 for iPSC-derived CMs: ECs: CFs as optimal. The type and amount of growth factors also has to be optimized as both the cells and growth factors will play a key role in determining the viscosity and other rheological properties of the bioink.

Another aspect of an ideal cardiac bioink involves the use of conductive materials as conductivity plays a major role in regulating cardiac functions [137]. Conductive polymers such as polypyrrole (PPy) [138,139] and polyaniline (PANI) [140,141] have already been used in vitro and in vivo cardiac applications, proving their biocompatibility. However, long-term effects of these conductive polymers and possibility of using them along with other growth factors are still to be explored.

#### 5.2.4. The Role of the Basement Membrane (BM) and Growth Potential

The basement membrane (BM) is a highly organized layer of the ECM consisting of a number of proteoglycans such as chondroitin sulfate proteoglycans and glycoproteins such as laminins, entactins, collagen type IV, fibronectin, and perlecan [142,143]. The role of cardiac BM in tissue engineering of cardiac tissues is an interesting and important area to be explored further. The importance of BM in the organogenesis of the myocardium cannot be understated, even with a limited knowledge of detailed interactions between the BM development, integrin expression and sarcomeric growth [144]. BM not only plays a key role in myocardial organogenesis but also in the regulation of cardiac electrical properties [145].

One of the key requirements in congenital cardiac surgery for an ideal cellular graft is its growth potential post-implantation; i.e., it should be viable and possess the ability to remodel and grow. To this end, bioprinting a developmental precursor that would serve as a template for in vivo organogenesis is a potential approach [146]. In other words, the developmental precursor approach involves bioprinting a rudimentary tissue structure with all the necessary biological cues including stem cells, growth factors, and their distribution. The bioprinted rudimentary structure, on implantation, will serve as a template for the organogenesis (growth and maturation into their adult counterparts as a complete organ). For example, Daly et al. [1] bioprinted a cartilaginous template that mimicked the geometry of a vertebral body, which, on subcutaneous implantation into the back of nude mice, supported the development of a vascularized bone organ containing trabecular-like endochondral bone with a supporting marrow structure. A correct representation of BM, in terms of its composition, structure, and function becomes necessary to successfully utilize the developmental precursor approach. Also, the in vitro or in vivo differentiation of stem cells into CMs pre- or post-printing mimics the sequential stages of embryonic cardiac development [130]. Hence, in addition to replicating the BM by bioprinting, incorporation of three important families of growth factors that play an essential role in cardiogenesis in the right quantities and form becomes necessary. The three growth factor families are the transforming growth factor β superfamily (specifically bone morphogenic proteins (BMPs)), fibroblast growth factors (FGFs), and the Wingless/INT proteins (WNTs) [129].

- There are several points to consider before this approach could be successfully used for cardiac tissue replacements:
- While the first and most-important step in the embryonic development of cardiac tissues namely the epithelial-to-mesenchymal transition (EMT) is understood, the complete developmental process of cardiac tissues such as valves is not fully understood yet [88]. Efforts to appreciate the missing links in the developmental process will go a long way in replicating the organogenesis using stem cells in vitro or in vivo.
- An ideal ratio of CMs:ECs:CFs for optimal myocardial viability from various stem cell sources has to be determined. A similar ratio should be established for the cardiac valves and great arteries.
- Perfect biomimicry of the cardiac BM by novel biofabrication strategies such as bioprinting should be explored.
- Sources of stem cells that are ethically non-controversial and easily isolated such as UCSCs and their differentiation into CMs and other associated cell lineages require further studying.
- An ideal bioink for bioprinting cardiac tissues, that would have the right type and concentration of cells, growth factors, and polymers/hydrogels that would promote the native developmental biology signals and cues needs to be formulated.

**Figure 7:**
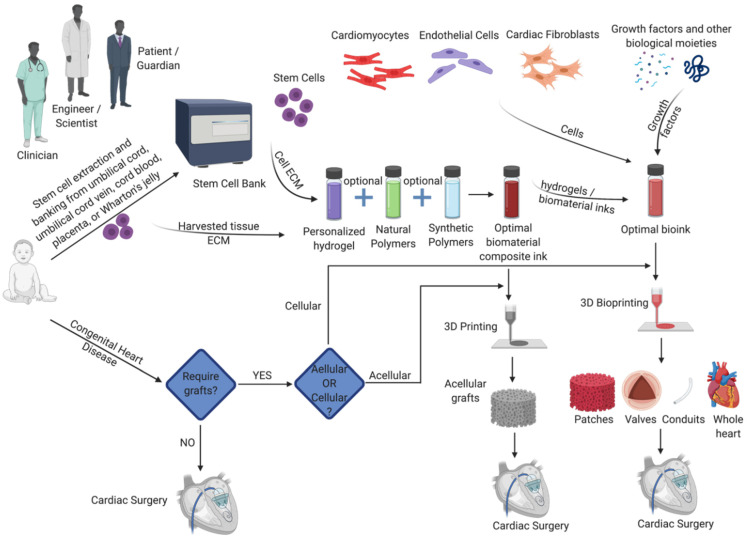
Process flow of biofabrication strategies for congenital cardiac surgery.

A potential process flow of biofabrication strategies for congenital cardiac surgery is given in Figure 7.

## 6. Discussion

Biomaterials presently available and employed in congenital cardiac surgery fall short of ideal expectations. Most implantation techniques and materials applied to babies derive from adult cardiothoracic surgery practices. Current biomaterials are designed to withstand the wear-and-tear of periodic distension and pulsatility but they underperform in small diameters, and they lack the potential of growth. While there has been huge progress in various domains of basic science of biomaterials, biofabrication, a significant gap still remains in translating those results into clinical applications.

**Figure 8:**
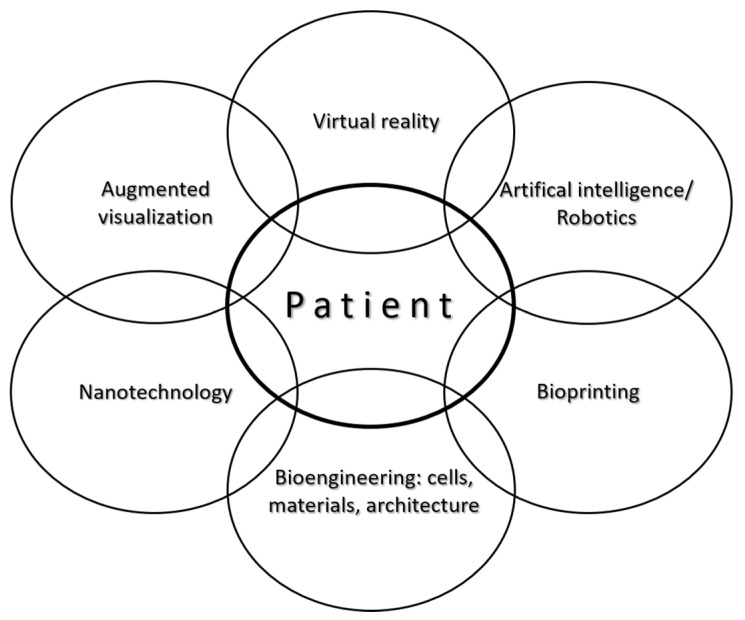
New methods and applications centered around the patient will revolutionize regenerative surgery.

Being a small but integrative discipline, with well-defined patient/recipient population, transparent pathways, standardized and primarily reconstructive procedures, closely-monitored outcomes, etc., congenital cardiac surgery naturally offers itself as a primary target for biofabrication and translational research. Reconstruction of congenital cardiac anomalies with viable, growing biomaterials could be a life changer for these patients, thus, biofabrication in congenital cardiac surgery is associated with enormous public health benefits. At present, most cardiac operations are aimed for and can restore the correct plumbing (i.e., segmental connections); however, patches, valves and/or conduits currently available are not of viable tissues and they do not contribute to tissue regeneration, so most patients face reoperations by simply outgrowing their 3D implants. Removal of the reoperation burden would be a financially estimable public health benefit let alone the associated improvements in quality-of-life of the individual patients. Advance in the outcomes of congenital cardiac surgery has been attributed to a problem-centered approach (e.g., primary complete repair), less-invasive techniques and much better materials and equipment. Still the aggression associated with open-heart surgery remains paramount. New modalities, e.g., augmented surgical visualization, virtual reality and artificial intelligence, along with the application of microrobots should reduce it and better biomaterials from nanotechnology, 3D-bioprinting and bioengineering of cells and tissue architectures are expected to revolutionize the shape of regenerative medicine and congenital cardiac surgery in particular (Figure 8).

The search for patient-specific, autologous (i.e., non-immunogenic), structurally sound, viable and growing implants for congenital cardiac surgery continues. Computer-aided design and 3D bioprinting can now produce individual implantable prototypes in many domains of reconstructive surgery [147,148]. Autologous tissues theoretically cancel out the problems of immunogenicity; however, they can prove structurally weak or non-viable at long-term (e.g., autologous pericardium valves). Allotransplantation attempts to meet best-match in for immunology and the biomechanical properties of the donor organ or tissue (e.g., heart-transplantation; immunology is a lesser problem with allograft valved conduits, patches); shortage of suitable donor hearts, especially for neonates remains insurmountable [149]. Xenotransplantation attempts to outwit evolution, but holds the promise of an unlimited pool of suitable organs and tissues [150]. Experimental results of cell xenotransplantation, e.g., islet or neuronal cells, are currently significantly better than those of organ xenotransplantation, illustrating Norman Shumway’s often-quoted phrase: ‘xenotransplantation is the future of transplantation, and always will be’ [150]. Table 3 summarizes the characteristics of xeno-, allo-transplantations and bioengineered tissues or organs.

Biofabrication also holds the promise of an unlimited supply of tissues and organs but without the hurdles of immunological issues for patients, especially babies with congenital heart disease. It is expected that the goal of a living and growing construct will be realized with the combination of evolving technologies in bioprinting and bioassembly, by perfecting composite biomaterials and autologous stem cells, inclusion of natural signaling cues and replaying developmental processes.

Both biofabrication and congenital cardiac surgery are relatively new disciplines. The blossoming branch of congenital cardiac surgery sprouted off from the trunk of surgery via cardiothoracic surgery some 60 years ago. The first congenital surgeons spent considerable time in the surgical laboratory and pathology museum. The next generation of surgeons may well come from scientific labs. ‘There is no way in which anyone can be an expert in all aspects of this enterprise. What will be required of the scientist of tomorrow is the ability to speak the language of other disciplines.’ The present paper is aimed to help the reader to become at least bilingual [151].

## Figures and Tables

**Table 1:** Essentials for acellular, cellular or composite constructs in congenital cardiac surgery.

Type of Construct	Clinical Scenarios	Specific Aspects and Anticipated Advantages
**Acellular**	Defect closure: ASD, VSD, etc. Intracardiac baffles: atrial separation (Mustard); intraventricular rerouting double outlet right ventricle (DORV); extracardiac: aortic arch repair	Structural robustness; flexibility; adaptability
**Cellular**	Regeneration of cardiac segments and structures: myocardium (univentricular heart), valves (pulmonary-, aortic atresia), vessels (pulmonary artery reconstruction)	Tissue integration, regeneration and growth
**Composite**	Conduits and valves to bridge gap (pulmonary atresia), augment hypoplastic and replace missing segments	Tissue integration, regeneration and growth

**Table 2:** Naturally derived vs. synthetic polymers.

	Naturally Derived Polymers	Synthetic Polymers
**Examples**	Gelatin, chitosan, fibrin, alginate	Polylactic acid (PLA), polyglycolic acid (PGA), their copolymer: PLGA; polydioxanone (PDO, PDS)
**Advantages**	General availability, excellent biocompatibility; autologous (fibrin); improved cell adhesion and cell invasion	Easily mass produced and sterilized; ability to regulate the microstructure, degradation rate, and mechanical properties
**Disadvantages**	Poor mechanical strength and rapid degradation rate (gelatin, fibrin); poor cell adhesion (chitosan); possibility of transmission of pathogens	Poor cell adhesion and cell invasion; decreased remodeling and growth; inflammatory response

**Table 3:** Characteristics of bioengineered materials, xeno- and allotransplantation.

	Bioengineered Tissues/Organs	Xenotransplantation	Allotransplantation
Availability, supply: present	Unresolved	Unresolved	Limited
Availability, supply: ideal-case scenario	Unlimited	Unlimited	Limited
Difficulty in manufacturing	High	High	n/a
Organ dysfunction due to donor brain death	n/a	n/a	Possible
Pathophysiological barriers	Numerous: “create life”	Numerous: “outwit evolution”	Moderate: “outwit immunology”
Biomechanical properties	Poor; improving	Adequate	Adequate
Growth possibility, endurance	Unresolved	Unresolved	Adequate with limitations
Immunology	n/a	Highly problematic	Problematic
Transmission of pathogens, sterility	Unlikely	Less possible	Possible
Cultural/ethical considerations, public opinion	Accepted	Mixed	Culturally variable
Regulatory considerations, infrastructure	Significant	Significant	Moderate
Costs	Moderate	High	Moderate-high

Abbreviation: n/a, non-applicable.

## Abbreviations

ASD	atrial septal defect
AVSD	atrioventricular septal defect
BM	basement membrane
BMSC	bone marrow-derived stem cell
CFs	cardiac fibroblasts
CHD	congenital heart disease
CMs	cardiomyocytes
ECs	endothelial cells
ePTFE	expanded polytetrafluoroethylene
HLA	human leukocyte antigen
iPSCs	induced pluripotent stem cells
LV	left ventricular
MAPCA	major aortopulmonary collateral artery
MPA	main pulmonary artery
MC	monocusp ventricular outflow patch
PDA	patent arterial duct
PV	pulmonary valve
RV	right ventricle
RVOT	right ventricular outflow tract
SIS-ECM	small intestine submucosa extracellular matrix
TAP	transannular patch
TGA	transposition of the great arteries
TOF	tetralogy of Fallot
UCSC	umbilical cord and placental stem cell
VSD	ventricular septal defect
